# The “Vulnerability” Discourse in Times of Covid-19: Between Abandonment and Protection of Canadian Francophone Older Adults

**DOI:** 10.3389/fpubh.2021.662231

**Published:** 2021-09-03

**Authors:** Martine Lagacé, Amélie Doucet, Pascale Dangoisse, Caroline D. Bergeron

**Affiliations:** ^1^Department of Communication, Faculty of Arts, University of Ottawa, Ottawa, ON, Canada; ^2^Faculty of Social Sciences, School of Psychology, University of Ottawa, Ottawa, ON, Canada; ^3^Department of Psychology, Université du Québec à Montréal, Montréal, QC, Canada; ^4^Division of Aging, Seniors and Dementia, Public Health Agency of Canada, Ottawa, ON, Canada

**Keywords:** older adults, pandemic, ageism, Francophone media, Canada

## Abstract

The Covid-19 pandemic has been particularly difficult for older Canadians who have experienced age discrimination. As the media can provide a powerful channel for conveying stereotypes, the current study aimed to explore how Canadian Francophone older adults and the aging process were depicted by the media during the first wave of the Covid-19 pandemic, and to examine if and how the media discourse contributed to ageist attitudes and behaviors. A content analysis of two French Canadian media op-eds and comment pieces (*n* = 85) published over the course of the first wave of the pandemic was conducted. Findings reveal that the aging process was mainly associated with words of decline, loss, and vulnerability. More so, older people were quasi-absent if not silent in the media discourse. Older adults were positioned as people to *fight for* and not as people to *fight along with* in the face of the pandemic. The findings from this study enhance the understanding of theories and concepts of the Theory of Social Representations and the Stereotype Content Model while outlining the importance of providing older people with a voice and a place in the shaping of public discourse around aging. Results also illustrate the transversality and influence of ageism in this linguistic minority context.

## Introduction

For the past four decades, researchers have studied the global media depiction of older adults^1^ and have found that the older population is typically underrepresented and portrayed negatively in Western cultures (1–8). Worldwide, older adults are described as part of a homogeneous and vulnerable group and aging is mainly discussed in terms of significant economic and demographic problems (the expression of the “gray tsunami” is one of many examples expressing these “problems”) (9–13). Older women and minority older adults experience a double marginalization by being even less represented in the media and are often portrayed as frail, unattractive, and invisible compared to older white men who are more often ^2^ described as experienced and powerful (7, 14–20). Social media has contributed to sharing similar types of messages and visuals, mostly reinforcing a negative discourse on aging (8, 21, 22). For example, at the beginning of the pandemic, an analysis of two media outlets in Spain revealed that older people were depicted negatively in the majority (71%) of cases (23). Along the same lines, a study by Xiang et al. (24) examining 82,893 global tweets related to older adults and Covid-19 from January to May 2020 found that 18% of daily tweets had ageist content, with the highest ageist content (53% of all content) published on March 11, 2020 (the day the World Health Organization officially declared that we were in a global pandemic).

Decades before the pandemic hit in Canada, studies had documented the prevalence of ageist stereotypes and attitudes in the health care sector (25), long-term care sector (26), workplace (27–31), through government policies, programs, and services (32), and in the media (33). A 2012 Canadian report revealed that 63% of people age 66 and older had been treated unfairly or differently because of their age (32). In addition, close to 80% of Canadians agreed that people ages 75 and older were often ignored compared to younger generations (32).

Although some researchers noted a trend toward positive representations of the older population, especially with the promotion of successful aging processes (7), negative perceptions of older people were exacerbated in the public discourse during the Covid-19 pandemic (12, 34–36). Language used such as “boomer remover” (35–37) suggested that the lives of older people were not as valuable as the lives of younger people. “Compassionate ageism,” also termed “caremongering” (38) or benevolent ageism (39), contributed to the association of chronological age with stereotypes of frailty, burden, and vulnerability, which undermined older people's agency and ability to care for themselves. Interestingly, while older adults were the focus of the news media and political decisions (40), their voices in the public discourse appear to be limited.

This study builds on an intersectional lens (41–43), whereby in addition to age, language is also considered an important identity factor that has been recognized as a potential target of negative stereotypes and discrimination. In Canada, Francophones are considered a linguistic minority; 21% of the country have French as their mother tongue (44). Findings from studies suggest that Francophones, as a linguistic minority, are at risk for language-based discrimination in all 13 Canadian provinces and territories, except in the province of Quebec (45, 46), resulting in limited access to services and resources (47). However, few studies have focused on Canadian Francophone older adults in terms of examining how they are portrayed in the public discourse, and precisely if and to what extent they may be the target of negative stereotypes.

Considering that public discourse—including the media discourse—can influence and partly shape one's identity (48), it is important to examine the nature of such discourse, in order to determine if indeed, it conveys or even strengthens stereotypical messages. This is especially important in the context of a global health crisis that has the potential to exacerbate negative age-based attitudes (49), as illustrated in the Global Report on Ageism (50). This paper examines how a minority group, namely Canadian Francophone older adults, were portrayed by the media during this global pandemic.

To frame this study, we turn to the Theory of Social Representations (51, 52) and the Stereotype Content Model (53, 54) which are core to understanding the role of public discourse and the nature of ageist stereotypes.

### Conceptual Framework

According to the Theory of Social Representations (51, 52), a social representation relates to a set of knowledge, beliefs, patterns of apprehension and action about a socially important object. In particular, a social representation refers to common sense knowledge that defines reality for the social whole and guides action and communication. Further, social representations exert an influence on individual representations. The media is a powerful producer of social representations that in turn partly shape norms and expectations regarding members of different social groups (55). In the case of age and aging, it is plausible to think that the collective representations produced and reproduced by the media as it relates to aging and older adults influence how individuals talk and think about their own aging process, how they relate to older adults and, more so, what they expect from older adults (56, 57).

Findings from previous studies suggesting that media representations promote stereotypes of burden, frailty and vulnerability around the aging process echo through the Stereotype Content Model (SCM) (53, 54). This model postulates that when individuals try to make sense of one another, they do so by relying on two basic dimensions related to social cognition, namely *warmth* (the extent to which a person can be trusted, is friendly, etc.) and *competence* (the extent to which a person is capable of accomplishing his or her goals, is assertive, etc.). When it comes to older adults, findings from studies relying on the SCM are consistent and converge toward the following: older adults are systematically categorized in the high end of warmth spectrum and in the low end of competency spectrum (58, 59). In turn, according to SCM, this descriptive stereotype of older adults' low competence and high friendliness evokes prescriptive emotions such as pity and sympathy (53, 54), which are actually reflected in compassionate ageist behaviors (60). Findings from North and Fiske (61) suggest that descriptive stereotypes pave the way to prescriptive stereotypes in that older adults are expected to behave in the way they are stereotypically portrayed and that derailing from such expectations may entail punishment or resentment. Recent studies conducted before and during the pandemic suggest that compassionate ageist attitudes are particularly expressed toward the oldest old (62), who, as argued by Higgs and Gilleard (63, 64), embody the most feared and marginalized aspects of aging and old age.

Relying on both the Theory of Social Representations (51, 52) and the Stereotype Content Model (53, 54), the current study will allow for further understanding of how older adults are portrayed in the media discourse in a time of global health crisis and, as such, determine if and how such a discourse subscribes to ageist descriptive and prescriptive stereotypes.

This study's research questions are as follows:

1. How were older adults and the aging process depicted by the media during the first wave of the Covid-19 pandemic in French Canada?
2. Did the media discourse contribute to ageist attitudes and behaviors?
  a. If so, how and to what extent?

## Materials and Methods

In order to answer the research questions, we relied on a descriptive content analysis of French-Canadian media, focusing on *La Presse* and *Le Devoir*.^3^ This choice was guided by the fact that these two newspapers are the largest Francophone newspapers in Canada, but also that they are most likely to focus on issues that matter to Francophone communities. Articles were coded (by two coders who are co-authors of this paper) using an iterative and cyclical process (65). Precisely, several rounds of initial coding were conducted to allow for themes, specific terms, information, and context to emerge. This process validated new insights and understandings, while informing coding and analysis. A collaborative approach was also used throughout the coding process to ensure inter-coder reliability. The descriptive content analysis has allowed for an exploration of the nature of the language used in the media during the first wave of the Covid-19 pandemic and identify language patterns as well as potential biases and stereotypes relating to older people and Covid-19.

### Selection of Articles

**Figure 1:**
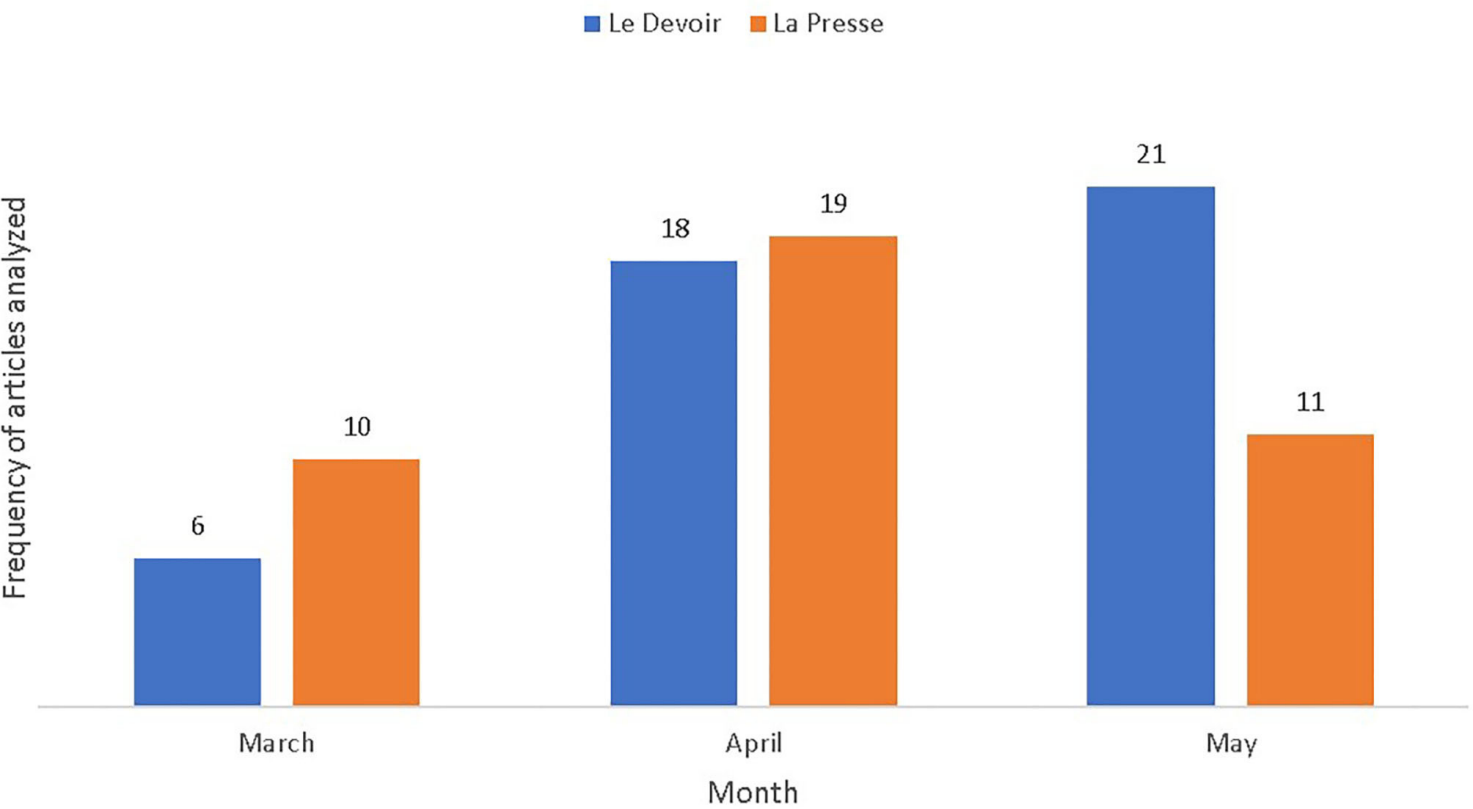
Distribution of articles analyzed by each journal across time.

An initial search of articles published in *Le Devoir* and *La Presse* from March 1, 2020 to May 31, 2020 was conducted. This specific timeframe was chosen as it corresponds to initial media reports warning about the spread of the Covid-19 virus within the North American continent, followed by restrictive measures put in place by the federal, provincial, and territorial governments and the lifting of the first wave confinement. Rather than news content, the selection focused on opinions and editorial pieces (Op-Eds), comment pieces and chronicles as these are important vehicles of expressions of divergent opinions and potentially powerful tools to promote public debates (66). To find relevant content from the two newspapers, we conducted a search using three international news source databases: Eureka, Factiva, and ProQuest, guided by a series of keywords such as: *Covid-19/Coronavirus* ^*^ *older people; older adults; seniors; elderly; grandparents; age; generations; baby-boomers (boomers), old, senior residences*, and *long-term care (long-term care facilities)*.^4^ As illustrated in Figure 1, the search yielded a total of 85 articles published from the beginning of March 2020 to the end of May 2020 in *La Presse* (*n* = 39) and *Le Devoir* (*n* = 46).

### Content Analysis

Each article was analyzed according to an initial list of codes developed by the research team. These codes examined: (1) how the media described older adults and the aging process during the Covid-19 pandemic; (2) if and how older adults were given a voice^4^ to express their views about the pandemic and its impact, and; (3) how older adults were positioned in the media in the face of the pandemic. From these three main codes, a series of categories were created based on previous studies on media discourse, aging, and ageism (67–70). Examples of categories related to how the media portrayed older adults and the aging process are as follows: active, healthy, lonely, vulnerable, knowledgeable, resilient, autonomous, obsolete. More so, the portrayal of older adults and aging were also categorized according to its tone, i.e., positive, negative or neutral. To determine if and how older adults were provided a space and a place in the media, special attention was given to whether articles: (1) were signed by older adults or associations of older adults; and (2) were included interview excerpts with older adults; with family members of older adults or caregivers of older adults. Finally, to assess how the media positioned older people in the face of the pandemic, three categories were identified: (1) older people are described as taking part, i.e., capable of taking part, in the fight against the virus; (2) older people are portrayed as individuals for whom *others* must fight for or protect; (3) older people are both fighting against the virus and victims that must be protected from it.

In order to maximize validity of the categories and ensure inter-coder reliability, two series of five articles were randomly selected and coded manually by two team members. These pilot tests enabled the thorough discussion and revision of our categories through an iterative process. Upon reaching a 70% level of agreement between the two coders, all 85 articles were organized in NVivo and were subsequently analyzed (71).

## Results

### Representations of the Aging Process and Older Adults

The aging process was discussed in 37 of the 85 articles. In the majority of these cases, aging was perceived as a process of decline, frailty, and death, as explained in this translated excerpt: “*Evidently, many long-term care residents swept away by the coronavirus would have died this spring, with or without the pandemic*” (Alexis Riopel, “Une hécatombe hors norme dans les CHSLD du Québec,” *Le Devoir*, April 25, 2020). Eight articles provided a more neutral overview of aging, by discussing both gains and losses. Only three media articles referred to positive aspects of aging, underlining the accomplishments and contributions of older adults to society, as illustrated in the following excerpt: “*If we were to trace back the history of Quebec over the last 80 years, we would discover the amazing accomplishments of the old and the wise*” (Pierre Paquette, “Claude Lafortune and Janette Bertrand, des modèles contre l'âgisme?”, *Le Devoir*, April 24, 2020).

Table 1 presents the frequency of terms used to describe older adults. The media described older adults mainly in terms of vulnerability and depicted them as alone and sick, in the process of losing or having lost their autonomy, or close to dying: “*The majority are a little confused and don't remember if they've had their breakfast. Imagine asking them if they've gone to a public place or if they were in contact with someone with Covid symptoms*.” (Magdaline Boutros, “Aller-retours à haut risque pour les infirmières en soins à domicile,” *Le Devoir*, April 16, 2020). It is interesting to note that the dominant media discourse around negative aspects of aging mainly related to older adults living in long-term care facilities who represent only 6.8 per cent of the older population in Canada (72) and who as we will see below, have no say in this framing.

**Table 1:** Frequency of keywords associated with older people.

**Connotation of terms (positive, negative, or both)**	**Terms used to describe older adults**	**Frequency of terms in the 85 articles**
Negative connotation	Vulnerable	39
	Alone, isolated	18
	Sick	11
	Death	20
	Obsolete, old fashioned	3
	Dependence, loss of autonomy	9
	Old	6
Neutral connotation	At risk	25
	Non-conformist	2
Positive connotation	Independence, autonomy	10
	Knowledgeable, history holder	7
	Grandparent (role)	11
	Active, healthy	3
	Resilient	3
	Tech savvy, technophile	2

The few articles that portrayed older adults in a more positive light counteracted this homogeneous and negative view of aging by focusing on older adults' good health and resiliency. These few articles were actually written by older adults: “*Obviously, such a crude, if not to say silly, statistic does not take into account the fact that many seniors are actually in better physical condition than many folks much younger*.” (Richard Lafaille, “De la coherence en temps de crise,” *Le Devoir*, May 9, 2020).

### A Place and Space for Older Adults in the Media During the Pandemic

The question of who spoke or was invited to speak in the media on issues around aging during the pandemic reveals that older adults were rather silent if not quasi absent from the discourse. Precisely, only seven articles out of 85 were authored by older adults, none by younger adults and most, not surprisingly, were written by columnists or editorialists of the newspaper (see Table 2). In the latter case, it is worth nothing that out of the 51 articles authored by columnists, only 12 included quotes from interviews with older adults. In the case of articles authored by older adults themselves, the focus was placed on healthy retirees' societal contributions: “*In doing so, we forget that healthy retirees play an important social role. Thus, the current pandemic has made it possible to realize that they form an important part of the volunteers in the various associations and groups that act in the communities. Without them, it is difficult for these organizations to fulfill their mandates*.” (Pierre Cliche, “J'ai 72 ans et je suis en bonne santé. Est-ce un tort?”, *La Presse*, May 30, 2020).

**Table 2:** Type and frequency of authorship.

**Author of the article**	**Number of articles by author type**	**Percentage (%)**
Journalist, editor, columnist	51	60
Citizen	15	18
Young citizen	0	0
Older citizen	7	8
Researcher or group of researchers	5	6
Association of seniors	0	0
Other	7	8
	85	100

### How Older Adults Were Positioned in the Media in the Face of the Pandemic

Echoing the depiction of aging mainly as a process of loss and decline as well as the predominant themes of vulnerability, loneliness and dependency of older adults, results reveal that the older population is described as not being able to take part in the collective fight against the virus, hence that others must protect them. Precisely, throughout the 85 articles analyzed, the majority of references to the positioning of older adults highlighted, here again, their vulnerability and inability to be part of those who fight the virus. Rather, they were portrayed as those for whom the rest of society must fight and make sacrifices for (see Table 3):

**Table 3:** Positioning of older people during the pandemic.

**Positioning of older adults in the context of COVID-19**	**Frequency of references in the 85 articles**
People we fight for and must be protected	33
People who take part in the fight against the virus	2
People who both take part in the fight but for whom the rest must fight for	5

“*For weeks, we self-imposed an exceptionally difficult confinement on all aspects of society to try to protect older Quebecers from Covid-19…”* (Christian Dufour, “Après le temps des vieux, le temps des enfants,” *La Presse*, April 30, 2020). In the few instances where older adults were portrayed as taking part in the fight against the virus—as much as the rest of the population did so, it is actually older adults themselves who conveyed such as message: “*Of course when we heard about the new restrictive measures announced by Premier Legault, we did not jump for joy. However, we are perfectly aware that by following such measures, we are contributing to the collective well-being. It is time to show solidarity!* (Constance Bennett, 73 years old, *La Presse*, March 18, 2020).

Interestingly, one comment piece suggested that above and beyond the role that older adults played during the pandemic, these were left out of the social debate around aging: “*In the aftermath of the CHSLD and seniors' residence scandal, a debate has arisen about aging in our societies. But judging by the way it is starting, I fear that it will unfold without the participation of the elderly. While more than 80% of the latter live at home independently, contribute in a thousand ways to the future of our societies and remain perfectly valid interlocutors*.” (Fernand Dansereau, “L'art de vieillir, selon Fernand Dansereau,” *Le Devoir*, May 25, 2020).

## Discussion

Using a content analysis of Canadian Francophone media, the current study examined how the process of aging and older adults themselves were depicted during the first wave of the Covid-19 pandemic and how this discourse might have exacerbated ageist attitudes and behaviors. Findings reveal that in most cases, both the aging process and older people were depicted negatively, through the use of terms such as “vulnerable,” “at risk,” “isolated,” “alone,” “disease” and “death.” More positive words such as “resilience,” “health,” and “bearer of wisdom” were far less used in the sample of media articles.

Further, the authors of these words were rarely older adults; very few articles were either signed by older adults or included their voices and perspectives (through the use of interview excerpts for example). The same can be said about older adults' relatives or caregivers who did not have a voice. In the case of older adults, most articles talked *about* these older adults, without providing any information related to their gender, ethnicity, nor any sociodemographic background. The combination of mostly negative language used to describe older adults on top of an impersonal and objectifying tone can contribute to the phenomenon of *othering* whereby older adults are viewed as members of an out-group that members of in-group keep distance from. As argued by Gendron et al. (73), this age *othering* process, conveyed through language undoubtedly contributes to ageism and negatively impacts older adults' health and social isolation.

Along the same lines, in the majority of the articles, there was a collective call *to fight for* older residents, to respect the public health measures in order *to protect* older people. In only a few instances, older people were portrayed as also having agency and being empowered to fight this virus for themselves and alongside others.

The depiction of the aging process and of older adults mostly in terms of decline and loss comes as no surprise as it falls in continuity with previous studies on media discourse (8, 73, 74). The media frames older adults as a homogenous group, and aging as a process of loss and decline both at the societal and individual levels.

According to the Theory of Social Representations (51, 52), it is plausible to think that the negative collective framing of aging and of older adults partly shapes the experience of aging at a *personal* level, translating, among young and older adults, into fear and anxiety about their own aging. It is interesting to note that while younger adults deal with anxiety about aging by expressing higher levels of ageist stereotypes and attitudes toward older adults (75), older adults psychologically dissociate themselves from members of their own age group as a self-protective strategy (76). In the current study, the very few articles signed by older adults seem to echo such a strategy: in these articles, while authors self-identified as older adults (by stating their chronological age for example), they also manifested a form of social distancing from other members of their age group whose health may be more precarious. This strategy helps to counter the media's ageist portrayals of older adults, but could also create a divide among older adults themselves whereby those who are fit and healthy distance themselves from those who are not, fostering intra-generational ageism. In a recent article, Higgs and Gilleard (64) actually argue that the Covid-19 pandemic has exacerbated a form of intra-generational divide between the third and the fourth age, the latter “*defined less by what it actually is than by what it is not. Its imaginary is shaped through its antithetical projection of a dependent old age and not the youthful, vital, healthy and successful aging that feature so much in the range of books and magazines promoting third age lifestyles.”* (p. 2). Hence, according to the authors, as old age and nursing homes represent the undesirable side of life, one that should be avoided, it should come as no surprise that these were the most negatively impacted by the pandemic. Along the same line, from a discourse perspective, findings of the current study suggest that older adults who have spoken in the media may have done so to actually express their sense of belonging (and wish to belong) to a third age culture and conversely, their resistance to the fate of the fourth age.

Findings from the current study also fall in line with the postulates of the Stereotype Content Model from Fiske et al. (54). This social cognition model suggests that older adults are most often perceived through the combination of high sociability ^*^ low competency; in other words, older adults are stereotypically depicted as kind individuals but who are not active agents of their lives. In the current study, the combination of high sociability ^*^ low competency actually translated in a majority of articles positioning older adults as frail and vulnerable people that cannot take part in the fight against the virus, but for whom the rest of society must do so. One of the negative outcomes of such age-based stereotypes, as argued by Fiske (53), is that it can lead to prescriptive ageist attitudes expressed through pity and sympathy toward older adults, taking the form of *benevolent* or *compassionate* ageism (38, 39). However, in times of global crisis such as the Covid-19 pandemic whereby resources may become more limited, it is plausible to think that benevolent ageism could turn into hostile ageism. In the very beginning of the lockdown, there were already signs of such hostile age-based attitudes in the social media world where the hashtag “*Boomer Remover”* circulated many weeks until removed (35–37). Pending the pandemic lasts for more months or years, a public debate could emerge opposing the wealth of the economy sacrificed because of the need to protect “vulnerable older adults”; such a debate that could indeed, pave the way for hostile ageism.

Paradoxically, age discrimination, negative age stereotypes, and negative self-perceptions of aging have a significant impact on the economy (77). A recent U.S. study estimated that one in every seven dollars spent on health care for the management of chronic conditions (e.g., cardiovascular disease, mental disorders, diabetes) was due to ageism alone (77). Ageism, as a social determinant of health (49), contributes to the prevalence of health conditions (77), worse physical and mental health (78) and premature death (79).

In summary, findings from this study suggest that the Covid-19 pandemic seems to have exacerbated collective manifestations of ageism precisely in the way the media discussed the aging process and older adults. Although positioned at the center of the crisis, older adults' perspective was quasi excluded from the media discourse in that others spoke on behalf of them. More so, the media mainly focused on the “problems” posed by an aging population and *de facto*, by older adults. Older adults were rarely portrayed as a source of power and support. It is worth noting that such ageist stereotypes were conveyed by Canadian Francophone media toward Francophone older adults. This suggests that ageism is a cross-cutting issue that shapes the media discourse where, even within a linguistic minority population of French Canadians, there are experiences of discrimination against the age minority status of certain social groups.

As the Canadian population continues to age, it is key to reflect on ways to counteract ageism and its negative impact. Paradoxically, these times of crisis may offer an opportunity to do so. First, we challenge the media to reach out to older adults, including residents of long-term care, to hear their perspectives on the pandemic. The more we seek to hear the voices of older people, the more their voices will be heard; in turn, the more place they will take in the public discourse, the more visible they will be in society, and the more their lives will be valued. Seeking their input may result in policymakers listening and considering the suggestions and solutions of those with more life experience in the management of the pandemic. Second, we urge older adults of all ages and the associations that represent them to share their stories using both traditional and social media and highlight the important contributions they are making during the pandemic. The more older people are seen and accurately represented in the media, the more they will encourage a greater number of older adults to stand up and show how they are actively fighting against the virus. Third, the results of this media discourse can be used to educate the Canadian population of the dangers of ageism and its severe repercussions in an aging society. Senior organizations, researchers, and the media are encouraged to collaborate in the creation of a national campaign against ageism.

The current study is not without limitations. First, the research focused on French-Canadian media and examined articles from only two newspapers in circulation. Results could differ or could be further validated if this study had included a greater number of Canadian francophone newspapers including *Le Droit* et le *Journal de Montréal*, or if it had also expanded its search to English Canadian media such as *The Globe and Mail* and the *National Post*. Considering the importance of language for a community's identity and culture, future studies should compare the media portrayal of older adults in both Francophone and Anglophone media. Another limitation is that the age of authors was unable to be ascertained with the exception of those that were volunteered by authors. Finally, the selected journals resulted in a small sample of 85 articles published from March to May 2020. Despite the sample size, this study provided an overview of the public discourse on aging and older people in times of Covid-19, specifically for Canada's linguistic minority. This research offered insight into how the Francophone public discourse, as articulated by the media, contributed to ageist stereotypes and behaviors toward Francophone older adults.

## Data Availability Statement

The original contributions presented in the study are included in the article, further inquiries can be directed to the corresponding author.

## Author's Note

The media is a powerful channel that may contribute to the spread of stereotypes and exacerbate discrimination. The current study aims at understanding how the Canadian media portrayed older adults during the first wave of the Covid-19 pandemic and the extent to which their discourse contributed to ageist stereotypes and attitudes, and in turn, negatively impacted older adults' health and well-being. Ageism is still rampant within North American culture and with the aging of the population, it is crucial to understand how and by whom it is conveyed, particularly in times of global crisis. Hence, this study advances knowledge as it relates to social representations of aging and ageism and the role that the media plays in shaping and framing such representations and, most importantly, how these can be re-framed.

## Author Contributions

All authors listed have made a substantial, direct and intellectual contribution to the work, and approved it for publication.

## Conflict of Interest

The authors declare that the research was conducted in the absence of any commercial or financial relationships that could be construed as a potential conflict of interest.

## Publisher's Note

All claims expressed in this article are solely those of the authors and do not necessarily represent those of their affiliated organizations, or those of the publisher, the editors and the reviewers. Any product that may be evaluated in this article, or claim that may be made by its manufacturer, is not guaranteed or endorsed by the publisher.

## Footnotes

^1^While older adults are typically defined as people ages 65 and older, for this study we purposely did not rely on any chronological cut-point to define what is an older adult so that the definition could emerge through the framing of the media discourse.

^2^All four authors of this article are fully bilingual (French-English); translations of quotes were drafted collaboratively.

^3^The keywords used in French were as follows: Covid-19/Coronavirus ^*^ personnes aînées (âgées); vieux; vieilles; grand-parents; âge; génération(s); baby-boomers (boomers); résidences pour aînés (pour personnes âgées); centres de soins de longue durée (centres d'hébergement de soins de longue durée).

^4^In most editorials or comments that included older adults' voices, the authors self-reported their age or began their article with statements such as “as an older woman myself.” Similarly, articles written by relatives or caregivers of older adults used statements such as “as the son of an older worker.” In other words, the point of view taken could easily be deducted by reading the text.
